# Investigating Aphasia Recovery: Demographic and Clinical Factors

**DOI:** 10.3390/brainsci14010007

**Published:** 2023-12-21

**Authors:** Georgios Papageorgiou, Dimitrios Kasselimis, Georgia Angelopoulou, Nikolaos Laskaris, Dimitrios Tsolakopoulos, Georgios Velonakis, Argyro Tountopoulou, Sophia Vassilopoulou, Constantin Potagas

**Affiliations:** 1 Neuropsychology & Language Disorders Unit, 1st Neurology Department, Eginition Hospital, Faculty of Medicine, National and Kapodistrian University of Athens, 11528 Athens, Greece; d.kasselimis@panteion.gr (D.K.); georginangel@gmail.com (G.A.); nikolaos.laskaris@gmail.com (N.L.); dimitris_tsolakopoulos@hotmail.com (D.T.); cpotagas@otenet.gr (C.P.); 2 Department of Psychology, Panteion University of Social and Political Sciences, 17671 Athens, Greece; 3 Department of Industrial Design and Production Engineering, School of Engineering, University of West Attica, 12241 Athens, Greece; 4 2nd Department of Radiology, General University Hospital “Attikon”, Medical School, National and Kapodistrian University of Athens, 15772 Athens, Greece; 5 Stroke Unit, 1st Department of Neurology, Eginition Hospital, National and Kapodistrian University of Athens, 15772 Athens, Greece; atounto@yahoo.gr (A.T.); svassilopoulou@gmail.com (S.V.)

**Keywords:** stroke, aphasia, lesion, language recovery, acute, chronic, naming, auditory comprehension, speech fluency

## Abstract

Post-stroke language recovery remains one of the main unresolved topics in the field of aphasia. In recent years, there have been efforts to identify specific factors that could potentially lead to improved language recovery. However, the exact relationship between the recovery of particular language functions and possible predictors, such as demographic or lesion variables, is yet to be fully understood. In the present study, we attempted to investigate such relationships in 42 patients with aphasia after left hemisphere stroke, focusing on three language domains: auditory comprehension, naming and speech fluency. Structural imaging data were also obtained for the identification of the lesion sites. According to our findings, patients demonstrated an overall improvement in all three language domains, while no demographic factor significantly contributed to aphasia recovery. Interestingly, specific lesion loci seemed to have a differential effect on language performance, depending on the time of testing (i.e., acute/subacute vs. chronic phase). We argue that this variability concerning lesion–deficit associations reflects the dynamic nature of aphasia and further discuss possible explanations in the framework of neuroplastic changes during aphasia recovery.

## 1. Introduction

Predicting the recovery of language functions following stroke is one of the most intriguing questions in the aphasia literature [1,2]. In recent years, several researchers have demonstrated the nebulous nature of aphasia recovery [3,4,5,6], even in the context of language rehabilitation [7]. Despite the variance observed among individuals with aphasia, it is generally accepted that there is a degree of language improvement after the onset of stroke, even in untreated patients [8,9,10]. However, it still remains challenging for clinicians to estimate the recovery of particular language functions, mainly because it is influenced by a variety of neural and behavior variables [10].

Advances in the field of neuroscience have clearly shown that the driving force of recovery and the reorganization of cognitive functions, namely neuroplasticity, is based on structural and functional changes in the brain, which eventually result in observable alterations in behavior; in several cases of post-stroke aphasia, the outcome of this process is the recovery of language functions, to varying degrees [11,12,13]. As for reorganization, the findings reported in the literature suggest that it occurs at different overlapping stages [14]. According to the general consensus, the first 3 months following stroke are considered to be the subacute post-stroke period [15]. In this particular timeframe, the allocation of language recovery is significantly higher [16,17]. In their study, Pedersen et al. [18] demonstrated that 80% of patients achieved stationary expressive language function within 2 weeks and 95% within 6 weeks. In contrast, recovery is rarely observed in the chronic phase, which spans from months to years post-stroke [19].

Concerning the improvement of language functions during the early stages of post-stroke aphasia, several demographic and clinical variables have been investigated as possible predicting factors [20]. Regarding the demographic variables, one of the core factors considered to be essential for prognosis is age at the time of stroke [15]. It is generally accepted that younger brains exhibit a greater degree of plasticity, and consequently, younger patients are more likely to recover than older patients [9,21]. With regard to sex, most studies have shown that there are no differences between men and women concerning aphasia recovery [9,22,23,24]. Education has been shown to be somehow involved in aphasia outcome, although it does not affect the recovery of language functions per se. Connor et al. [25] have found that an increased aphasia severity may be associated with a lower educational level, but not with the rate of recovery. In line with the latter finding, other researchers have argued that years of formal schooling have no impact on post-stroke language recovery [26,27].

In general, clinical variables are considered to be more reliable predicting factors compared to demographic variables. In recent years, an increasing number of studies have investigated the negative effect of larger lesions on the recovery of post-stroke aphasia. Although there is a study by Lazar et al. [26] suggesting that the lesion size does not predict language recovery, most studies have confirmed an inverse relationship between recovery and lesion size [28,29,30]. It should be, however, noted that aphasia recovery is not solely determined by lesion extent; the lesion locus is also a critical factor. A small lesion in the perisylvian area is more likely to have an impact on aphasia severity and recovery, while a large lesion in other brain areas may have minimal effects on different language modalities [31,32,33]. For example, lesions in the superior temporal gyrus (STG), particularly the posterior portion, have been associated with poor language recovery [34,35]. Accordingly, a preserved left superior temporal gyrus and intact basal ganglia have been identified as important factors contributing to satisfactory recovery [33,36]. It has been also suggested that cortical lesions tend to result in more severe aphasia compared to subcortical lesions, indicating that aphasia associated with subcortical lesions has a more favorable prognosis [37].

It is a common view that the observed spontaneous recovery may depend on the specific language function or aphasia type examined; for a review, see [5]. From that perspective, a significant number of studies have examined whether classic aphasia syndromes may exhibit reliable prognoses. In their study, Kertesz and McCabe [19] found that patients diagnosed with anomic, transcortical or conduction aphasia demonstrated excellent spontaneous recovery, while patients characterized as having Broca’s or Wernicke’s aphasia had a worse range of language outcomes. It is not uncommon for patients to be initially classified into one specific syndrome and then evolve into another one, even within weeks [38]. However, the consistency and reliability of the classic model of aphasia has been heavily criticized in recent years; see, for example, [39,40]. It has been argued that in order to overcome this issue in language recovery studies, they should focus on the dynamic process of language following stroke, instead of “evolving” syndromes in the different phases of aphasia. Indeed, one of the most important facts about language recovery after stroke is that it has been described as a non-linear process, while differences in recovery patterns have been associated with lesion variables and their impact on language functions [41].

The disentanglement from the restraints of taxonomies allows for the investigation of aphasia recovery according to a deficit-based approach. Regarding single-word comprehension, Selnes et al. [42] assessed patients at 1, 3 and 6 months following stroke and indicated that patients with damage to the posterior superior temporal cortex had better outcomes. Lesions affecting the supramarginal and angular gyri have been reported to be important predictors of the recovery of complex auditory comprehension, which has been shown to improve during the earlier stages of post-stroke aphasia [43]. Similarly, Selnes et al. [44] have demonstrated that a poor sentence comprehension outcome at 6 months following stroke was observed after lesions in the left posterior superior temporal and supramarginal gyri. Concerning the speech output of patients with aphasia, there is substantial evidence that verbal communication, speech rate and naming skills are the last to recover during the first 6 months subsequent to stroke, especially if there is a lesion in the inferior frontal gyrus [45]. Hillis and colleagues [6] have reported that damage to the left posterior part of the superior temporal gyrus may negatively affect the degree of recovery of naming skills in acute and chronic patients with aphasia. It should be, however, noted that predicting the recovery trajectory of language based on lesion location is challenging due to the substantial variability in outcomes, even between two individuals with similar lesions [46,47].

The main goal of the current study is to explore different demographic and clinical factors that better predict language outcomes in patients with post-stroke aphasia. As stated before, in previous studies of spontaneous language recovery, there are widely reported inconsistencies regarding the traditional aphasia syndrome analysis and its association with recovery patterns [10]. To that end, in the present study, we did not categorize patients into classic aphasia subtypes, but instead we mainly focused on the recovery of three specific language functions, as well as their lesion correspondence: speech rate, auditory comprehension and naming. Although scarce, there are studies which, contrary to the commonly held belief [5], indicate that lesion–deficit associations could progressively change across different stages of aphasia [48,49]. Based on that notion, we aimed to investigate whether the deficit-dependent course of recovery and its relationship with lesions demonstrates different patterns in the acute/subacute and chronic phases of aphasia.

## 2. Materials and Methods

### 2.1. Participants

Language and brain imaging data were analyzed from 42 patients (14 women) with acquired aphasia due to a single left hemisphere stroke. All participants were right-handed and native speakers of Greek, 23–84 years old, with 6–18 years of formal schooling. All patients underwent neurological examination and no vision or hearing deficit was reported by the neurologist. Equally, 12 patients reported receiving language therapy between the acute and the chronic phase. The patients were assessed at two testing times by clinical neuropsychologists and one speech language therapist, all of whom had robust expertise in aphasia assessment (authors G.P., D.K., G.A. and D.T.). The mean time post-onset for the first examination was 18.68 days, and for the second, it was 305.53 days. Informed consent was obtained from all patients prior to participation. For patients’ demographic information, please see Table 1.

### 2.2. Neuroimaging Data

**Figure 1:**
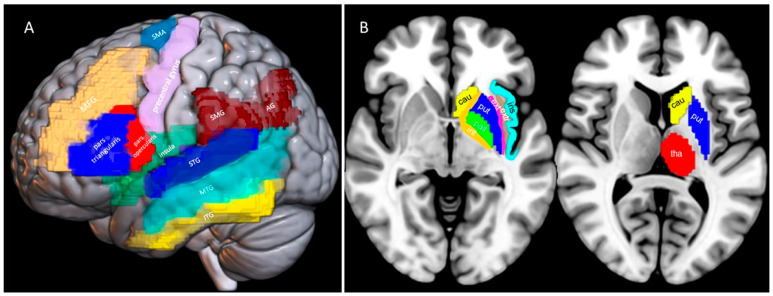
(**A**): The basic lesion loci identified for each patient (see Section 2 Materials and Methods). Middle frontal gyrus (MFG), inferior frontal gyrus (pars triangularis and pars opercularis), precentral central gyrus, supplementary motor area (SMA), insula, inferior parietal lobule (supramarginal and angular gyrus), superior temporal gyrus (STG), middle temporal gyrus (MTG) and inferior temporal Gyrus (ITG). Three-dimensional (3D) model was built from AAL Atlas ROIs normalized in SPM12 space, using MRIcroGL. (**B**): The basic lesion loci identified for each patient (see Section 2 Materials and Methods). Caudate (cau) in yellow, putamen (put) in blue, pallidum (pall) in green, thalamus (tha) in red. Three-dimensional (3D) model was built from AAL Atlas ROIs normalized in SPM12 space, using MRIcroGL. White matter areas are manually highlighted; internal capsule (int) in orange, external capsule (ext) in pink, extreme capsule (extr) in brown. Insula cortex (ins) is highlighted in light blue.

**Figure 2:**
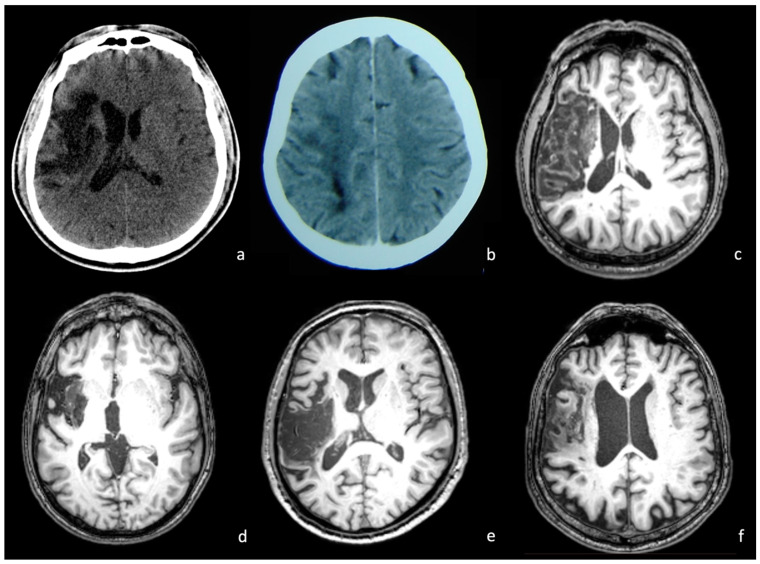
Examples of CT (**a**,**b**) and MRI (T1w) (**c**–**f**) axial images of stroke patients, based on which the neuroradiologists identified lesion locus. Each scan corresponds to a different patient.

Structural imaging data (non-digital CT and/or MRI scans) were obtained for each patient (only CT for 18 patients, CT and MRI for 18 patients, only MRI for 6 patients). For 35 patients (83.3% of the total sample), imaging was obtained within the first 10 days post-stroke. There were 3 patients for whom imaging was obtained 11 to 50 days post-stroke. For the remaining 4 patients, imaging was obtained more than 2 months post-stroke. The lesion sites were identified and coded by two independent neuroradiologists for 16 predetermined left hemisphere areas. Areas of interest included the inferior frontal gyrus (pars triangularis and pars opercularis), the middle frontal gyrus, the precentral gyrus, the supplementary motor area (the posterior part of the superior frontal gyrus), the inferior temporal gyrus, the middle temporal gyrus, the superior temporal gyrus, the insula and the inferior parietal lobule (consisting of the angular and supramarginal gyri). The surface areas are shown in Figure 1A. The thalamus, the head and tail of the caudate nucleus, the putamen and the globus pallidus were the deep gray matter structures evaluated. White matter areas were also reviewed, including the internal capsule (localized between the thalamus and pallidum), the external capsule (between the putamen and claustrum) and the extreme capsule (between the claustrum and insular cortex). Deep structures are shown in Figure 1B. The definition of each anatomical area was based on well-known landmarks already available in the literature [50,51,52]. The selection and coding of the aforementioned areas was based on the previously reported methodology [39]. The total number of affected cortical and subcortical areas served as an index of the lesion extent (lesion score) (as described in [53,54]). All imaging studies were evaluated by both neuroradiologists in order to verify stroke and to exclude alternative diagnoses, as well as to describe the lesion localization (see Figure 2).

### 2.3. Procedures

To assess language deficits, we used the Boston Diagnostic Aphasia Examination-Short Form (BDAE-SF) [55] adapted into Greek [56] and the Boston Naming Test (BNT) [57] standardized in Greek [58,59]. The BDAE-SF [56] is a speech and language battery that includes separate subtests that measure speech fluency, naming, repetition and comprehension. In the present study, specific BDAE-SF subtests were used: the auditory sentence comprehension subtest and the oral expression subtest. In the auditory sentence comprehension subtest, the patient is asked to execute a command spoken by the examiner. The stimuli range from simple (e.g., a two-item command, such as “show me the ceiling, and then the floor”, where the patient receives a maximum of a two-point score) to more complex commands (e.g., a five-item command, such as “tap each shoulder twice with two fingers, while keeping your eyes closed”, where the patient receives a maximum of a five-point score). We summed the total score of these auditory comprehension categories in order to create the BDAE-SF auditory subscale. In the oral expression subtest, the patient is asked to describe the Cookie Theft Picture. In the present study, the story was recorded and then the speech rate (words/minute) was calculated for each patient by two independent judges (inter-rater consistency: r = 0.98, *p* < 0.001).

## 3. Results

**Figure 3:**
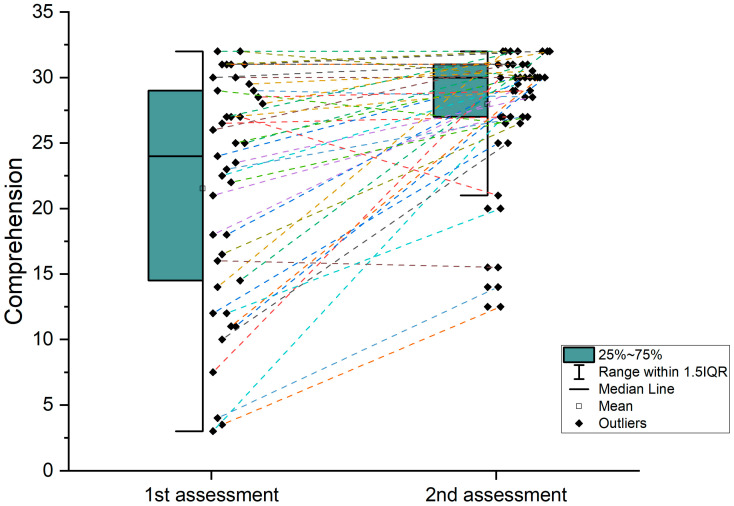
Individual data on the recovery of comprehension from first to second assessment. This graph depicts the participant comprehension scores from the two assessments. In the first assessment, the box plot reveals a broad range of scores, all within a 1.5 interquartile, suggesting the high variability of comprehension scores. In contrast, the second assessment’s box plot is shorter, indicating a more uniform level of scores among participants, although there are a few outliers. The median for the second assessment is of higher value than of the first assessment, implying an overall improvement. Also, the mean score shows a slice increase, indicating a slight improvement in comprehension.

**Figure 4:**
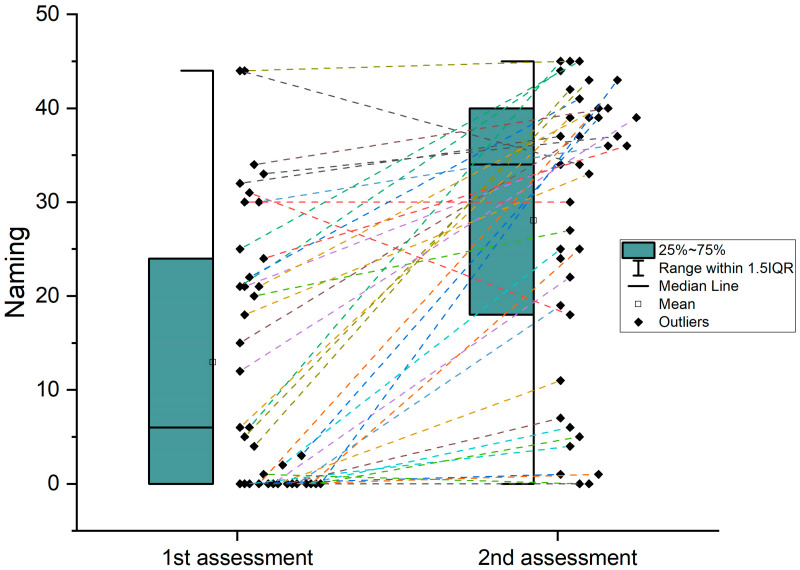
Individual data on recovery of naming from first to second assessment. This graph depicts the scores in naming tests from first to second assessment. In the first assessment, scores show a range of values beginning from zero up to 45 while a lot of participants have scored near or equal to zero. The box plot of the second assessment’s scores is considerably shifted upward, indicating a notable improvement (also represented by median and mean scores) and in general a wider spread of scores with values more than zero.

**Figure 5:**
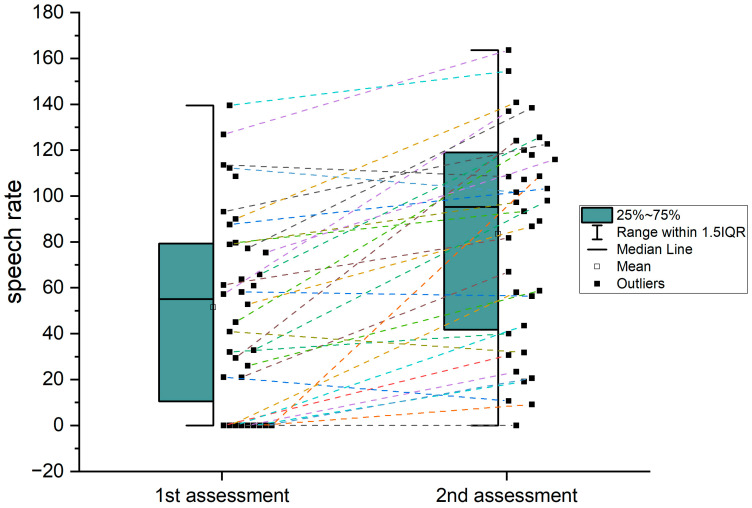
Individual data on recovery of speech rate from first to second assessment. This graph depicts an overview of participant’s speech rate progression between first and second assessment. The first assessment box plot indicates a lower mean and median values and also a concentration of values near zero. In contrast, for the second assessment, the box plot mean and median are higher values, and also the interquartile range is higher, indicating a greater variability in speech rates among participants but with almost no values near or equal to zero. As an overall evaluation, the speech rate has a general trend of improvement between the two assessments.

Comparisons using paired-sample *t*-tests between the acute and chronic phase revealed an improvement in performance for all language indices, meaning the Boston Naming Test [t(37) = −6.294, *p* < 0.001], the comprehension BDAE-SF subscales [t(40) = −5.584, *p* < 0.001] and the speech rate [t(32) = −6.137, *p* < 0.001] (see Table 2 and Figure 3, Figure 4 and Figure 5). Comparisons using Mann–Whitney U non-parametric tests to compare recovery in all three language domains between patients who received therapy and those who had no therapy history yielded null results. This study was not aimed at assessing any particular intervention program. Therefore, it should be clarified that the 12 patients who reported receiving therapy most probably followed different intervention programs (in terms of approach, intensity/frequency and total duration), which were not monitored by our team. Accordingly, the non-parametric comparisons are reported here just to show that the two subgroups did not differ in terms of recovery, and thus any observable change cannot be attributed to the confound of therapy. On the other hand, since the 12 patients followed heterogenous intervention programs of variable intensity and duration, and taking into account that the relevant available information was limited, the above reported null results are not generalizable and should not be considered evidence against the efficacy of speech and language therapy in aphasia. That is why these results will not be discussed further.

Regression models were conducted to investigate whether score differences between the testing times for the BNT score, speech rate and comprehension BDAE-SF subscales could be predicted by lesion extent, age of onset or years of formal schooling. Thus, separate multiple regression models were created, using the BNT score, speech rate or comprehension BDAE-SF subscales as dependent variables in each and introducing the lesion extent, age of onset and years of formal schooling as factors. The results revealed that none of the three models was significant, while any of the beta values of the aforementioned factors could significantly predict score differences between the testing times for the BNT score, speech rate or comprehension BDAE-SF subscales.

To investigate the possible role of specific ROIs (extreme capsule, inferior frontal gyrus, inferior parietal lobe, superior temporal gyrus and middle temporal gyrus) in certain aspects of language, and whether it differentiates the acute from the chronic phase, we conducted independent sample *t*-tests, separately for each phase of stroke (acute and chronic), each time using a binary variable of lesion–non-lesion ROI as the independent variable and language performance as a dependent variable. Thus, a total of 30 independent sample *t*-tests were performed, while FDR-adjusted *p*-values were then applied for corrections for multiple comparisons. The rationale of selecting these specific ROIs was based on previous work by our group [54], but also on published evidence illustrating the importance of these areas for language, and especially the role of these lesion loci in aphasic disturbances; see, for example, [33]. We present below our main findings.

Inferior Frontal Gyrus and Extreme Capsule. The results indicated that patients with lesions in the extreme capsule (EmC) have a significantly worse performance in comprehension compared to those with an intact EmC [t(39) = 2.249, *p* = 0.030], only for the acute phase. Also, patients with lesions in the inferior frontal gyrus (IFG) have a significantly worse performance in speech rate compared to those with an intact IFG [t(34) = 2.198, *p* = 0.035], only for the acute phase. No other comparison revealed significant results.

Inferior parietal lobe. The results indicated that patients with lesions in the inferior parietal lobe (IPL) have a significantly worse performance in the Boston naming test compared to those with an intact IPL [t(37) = 2.962, *p* = 0.005], only for the acute phase. No other comparison revealed significant results.

Superior temporal gyrus. The results indicated that patients with lesions in the superior temporal gyrus (STG) have a significantly worse performance in comprehension compared to those with an intact STG [t(38.98) = 2.425, *p* = 0.02], only for the acute phase. No other comparison revealed significant results.

Middle temporal gyrus. The results indicated that patients with lesions in the middle temporal gyrus (MTG) have a significantly worse performance in the Boston Naming Test compared to those with an intact MTG [t(37) = 2.518, *p* = 0.016] and in comprehension [t(13.46) = 2.796, *p* = 0.015], only for the chronic phase. No other comparison revealed significant results.

**Figure 6:**
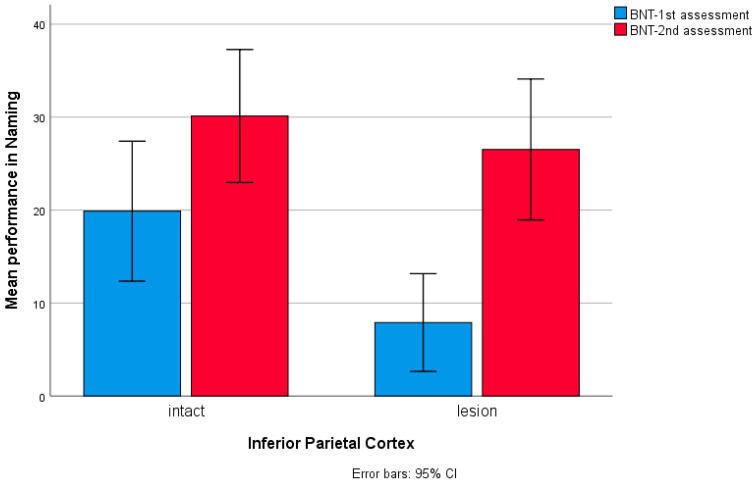
Bar chart for patients’ performance in Boston Naming Test at two testing times in relation to lesions in the inferior parietal cortex, with 11 patients having a lesion affecting the IPL.

**Figure 7:**
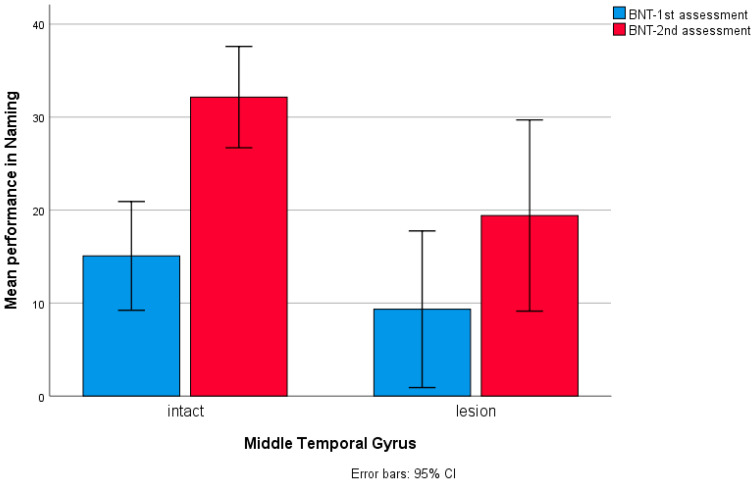
Bar chart for patients’ performance in Boston Naming Test at two testing times in relation to lesions in the middle temporal gyrus, with 13 patients having a lesion in the MTG.

**Figure 8:**
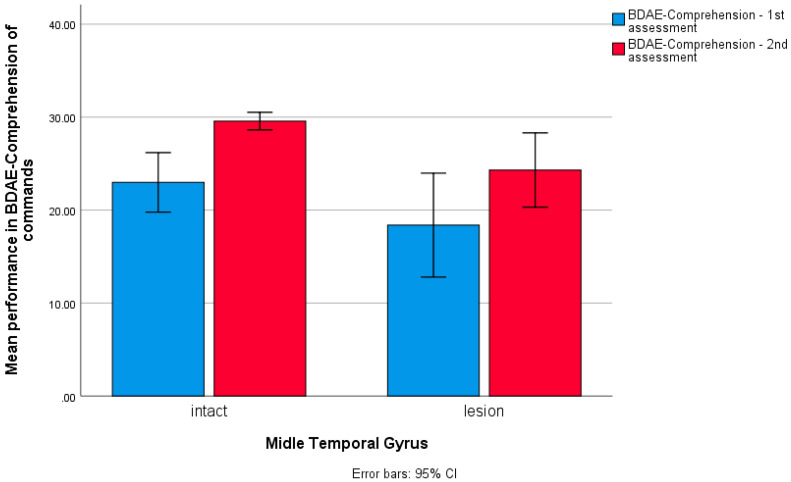
Bar chart for patients’ performance in the BDAE Comprehension subscale at two testing times in relation to lesions in the middle temporal gyrus, with 13 patients having a lesion affecting the MTG.

Corrections for multiple comparisons using false discovery rates (FDRs) revealed that the only comparisons that survived were those for the IPL as a predictor for performance in the Boston Naming Test in the acute phase [t(37) = 2.962, *p*_adjusted_ = 0.03192], and the MTG as a predictor for performance in the Boston Naming Test [t(37) = 2.518, *p*_adjusted_ = 0.048729] and in comprehension [t(13.46) = 2.796, *p*_adjusted_ = 0.048729] in the chronic phase (see Figure 6, Figure 7 and Figure 8).

**Figure 9:**
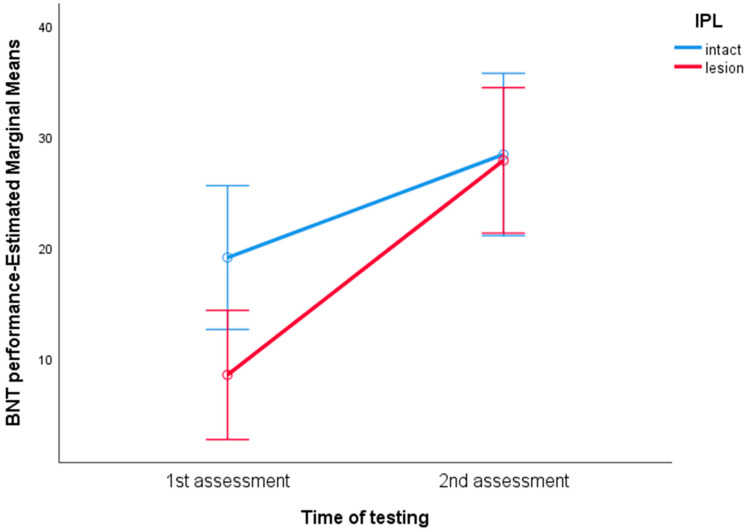
Estimated Marginal Means for BNT performance in the acute/subacute and chronic phase. The red line depicts the course of patients with lesioned IPL. The blue line depicts the course of patients with intact IPL. Error bars show 95% CI. The graph illustrates the interaction between time of testing and lesion in the IPL.

We then conducted Repeated Measures Analyses of Covariance (henceforth RM-ANCOVA) to assess possible interactions between the time of testing and lesion loci, while controlling for the lesion extent. The variables entered into these statistical models were selected on the basis of the aforementioned *t*-tests’ results. In the first RM-ANCOVA, the BDAE comprehension performance was entered as a within-subjects variable (i.e., patients’ scores at first and second assessment), MTG lesion as a between-subjects factor (binary variable: lesion vs. intact) and the lesion score as a covariate. No significant interaction between the time of testing and MTG lesion was found. In the second RM-ANCOVA, the BNT performance was entered as a within-subjects variable (i.e., patients’ scores at first and second assessment), MTG lesion as a between-subjects factor (binary variable: lesion vs. intact) and the lesion score as a covariate. No significant interaction between the time of testing and MTG lesion was found. In the third RM-ANCOVA, the BNT performance was entered as a within-subjects variable (i.e., patients’ scores at first and second assessment), IPL lesion as a between-subjects factor (binary variable: lesion vs. intact) and the lesion score as a covariate. There was a significant interaction between the time of testing and IPL lesion [F(1.35) = 4.538, *p* = 0.04] (see Figure 9).

## 4. Discussion

The primary aim of this research was to investigate the potential influence of demographic- and lesion-related factors on language recovery following stroke. Our study provides evidence that patients with aphasia, as a group, demonstrated improvement in speech rate, auditory comprehension and naming abilities. This is consistent with previous reports which indicate that usually the majority of people with aphasia following left hemisphere stroke perform better in language tasks within the first three to six months with little change thereafter [60].

In our sample, contrary to the conventional assumptions [8], traditional demographic variables such as age, sex and educational level did not significantly impact the recovery process of different language modalities. Concerning the potential influence of sex on language recovery, it has been theorized that men and women may exhibit different activation patterns of language distribution across the left and right hemisphere [61]. Thus, females could possess a more balanced distribution of language functions between both hemispheres comparing to males [62]. As a result, they might experience less impairment from left hemisphere strokes due to supplementary language support from the right hemisphere. However, our study did not confirm this assumption as there were no significant differences between genders in terms of aphasia recovery. Other studies in the field of aphasia validate our findings [9,24], whereas one study reported a slightly greater improvement in males [63]. This inconsistency regarding the impact of sex in language recovery could be probably attributed to recent studies which report that in controls, sex differences in brain structure or function, although present, do not necessarily reflect variations in language performance [64,65]. The emergence of sex differences then could depend on the modality of the language task, such as perception or speech fluency, and not specifically on the different language lateralization of brain structure per se [66].

Regarding the age of stroke onset and language recovery, our results did not provide evidence of a relationship. Most clinicians that attempt to estimate the language course of people with aphasia tend to believe that younger or more educated patients will demonstrate the greatest bulk of recovery [67]. The theoretical underpinning of investigating age as an important factor is based on the notion that the structural and functional reorganization of language following stroke takes place more readily in a younger nervous system [48]. However, concerning the influence of the aforementioned demographic variables, there are studies which indicate an opposite pattern. Ferro and Madureira [68] and De Renzi and Ferrari [69] have reported patients with non-fluent aphasia to be younger than those with fluent aphasia. One line of research also suggests that older individuals may possess greater cognitive resources that facilitate recovery, while younger individuals, despite the fact that they exhibit a greater degree of neuroplasticity, may face more important pathophysiological challenges related to the severity of stroke [15]. Our findings align with recent research which highlights that age and educational level are complex variables that encapsulate other important constructs in determining recovery outcomes [60]. For example, cognitive reserve, pre-existing language abilities or learning disabilities, socio-economic status and cultural influences may be pivotal in the language recovery even of two patients of the same chronological age. As this variety of confounds make this area particularly demanding to clarify, future research should make adjustments and implement careful planning of experimental designs in order to draw any valid conclusions regarding the impact of age, sex and education on language recovery.

Several studies in people with aphasia have explored the reliability of lesion location or lesion extent as credible predictors of language recovery, in the acute [70] or chronic phases [71]. In our study, regression analyses indicated that lesion extent did not predict speech rate, auditory comprehension or naming recovery. This finding is confirmed by another line of research which supports there being no clear association between the lesion extent and compensation of language [47], although there are studies which indicate an opposite pattern [9,18]. However, it has been argued that other potential explanatory factors which include the type of stroke or the amount of white matter integrity could influence the nature or the size of lesion extent and thus its impact on language recovery [15,20]. For example, it has been reported that hemorrhagic strokes lead to a greater lesion extent compared to other types of stroke [72]. In our study, however, there were no data regarding the type of patients’ stroke, which in part could explain its low predictive value in language recovery. We should also note that we used an index of the lesion extent (i.e., the sum of damaged areas, as indicated by 16 bivariate variables—see Section 2 Materials and Methods), which could be characterized as less reliable compared to the quantified lesion size extracted using volumetric methods applied to digital MRIs. Moreover, it is important to take into consideration that different studies have used various lesion variables, such as lesion size, lesion volume or lesion load, all of which, despite their differences, refer to the term “extent” [6,19]. As stated before, there are studies which indicate that lesion size or lesion volume could differently influence language recovery [48]. Future attempts should focus on the decoding of lesion extent as an important predicting variable and deconstruct it into simpler structures, while investigating their effect on language reorganization after stroke.

Contrary to the low predictive value of lesion extent in aphasia recovery, our findings have demonstrated that lesion location may differentially affect specific language functions in the acute/subacute and chronic phases. More specifically, in the acute/subacute phases, patients with lesions in the inferior parietal lobe have demonstrated a worse performance in naming, while lesions in the inferior frontal gyrus were associated with an impaired speech rate. The inferior parietal lobe has been reported to be an important hub of the dorsal language stream [73], which mainly supports phonological processing and word-finding functions, both essential in confrontational naming [6,74]. Additionally, it has been supported that the BNT test requires the naming of a variety of tools [75]. Thus, lesions in the inferior parietal lobe could lead to naming deficits, as this area has been linked to the manipulation and word finding of hand-used tools [76]. On the other hand, a substantial amount of evidence has established the fundamental role of the inferior frontal gyrus in the speech fluency of acute patients with aphasia, which is validated by our findings well [10,15]. Regarding auditory comprehension, we have demonstrated that it was impaired in the acute phase when the superior temporal gyrus was damaged, an area traditionally associated with understanding spoken language [77]. These findings align with neuroanatomical models which propose that this region is part of the ventral stream and critical for language processing and integration [78]. In line with these results, damage to the EmC was also associated with impaired comprehension in the acute phase. The EmC has been shown to be an integral component of the ventral stream, which has been argued to support semantic processing [73]. In addition, it has been reported that extensive lesions destroying language-related white matter tracts including the EmC will most probably result in comprehension impairment [79,80]. It should be noted that a selective lesion affecting specific components of the ventral stream, such as the EmC and pars triangularis, has been shown to result in specific deficits in lexico-semantic processing and active selective controlled retrieval [81]; it could be thus argued that a lesion in the EmC could, in synergy with other lesion loci (as is true for our patients, since they had much more extensive lesions), result in impaired underlying cognitive mechanisms, which in turn could affect the observed deficits in auditory comprehension. Interestingly, in our research, the influences of the aforementioned lesion loci were not observed in the chronic phase. Although the relevant evidence is scarce [48,82], it has been suggested that lesions in the acute phase could provide critical information for the brain–language association, while chronic lesions are more explanatory for the mechanisms that dictate language compensation. Τhis is in accordance with our findings, which indicate that patients with lesions in the middle temporal gyrus exhibited naming and auditory comprehension deficits only in the chronic phase. This could be attributed to a core principle of the brain, neuroplasticity, which allows the reorganization of cognitive functions following brain damage, including language [13,83]. It has been suggested that the reorganization of language involves different structural and functional processes which exhibit different time courses and include reperfusion, diaschisis and recovery from structural disconnection [73]. In their case series study, Jarso and colleagues [84] report that the reorganization and the recruitment of other areas could also depend on the particular language task examined, in addition to the site and size of lesion and time from the stroke onset. Thus, depending on the time of the assessment, there could be different lesion–language relationships in the acute or chronic phases, mainly because other brain regions and compensatory mechanisms could have a more prominent role in long-term aphasia recovery. This is further confirmed by recent studies which highlight that patients with aphasia can exhibit improvement in language even after 6 months following stroke [85]. Future studies could explore these compensatory mechanisms more thoroughly and identify additional brain areas involved in chronic phase language recovery.

One interesting finding that needs further exploration and validation (or to be disproved) by future studies is that of the differential involvement of the IPL lesion loci at the two testing times. The inferior parietal cortex has been shown to be generally involved in some aspects of language processing [86] and has been considered to be (at least partly) an anatomical component of Wernicke’s area by many researchers [40]. It should be, however, noted that there has been evidence against the involvement of the IPL in naming [87], and there have been several studies suggesting that the prefrontal and temporal cortices are fundamentally crucial for supporting the subprocesses underlying the ability to name a visually presented stimulus [33,88,89]. Accordingly, the lesion–deficit association pattern observed only in the acute/subacute phase (i.e., the transient negative effect of IPL lesions on naming ability) could be explained in terms of diaschisis [90] within a hodotopic framework for clinicopathological correlations as proposed by Catani and Ffytche [91]. Although this notion could be supported by reported evidence of the involvement of IPL lesions in naming impairment in acute patients with aphasia [92], there are also published data suggesting that there are long-term effects of such lesion sites in chronic stroke survivors. Left lesions affecting the inferior parietal areas have been recently shown to be, among other lesion sites, of predictive value concerning aphasia severity [93] and particularly naming [94] in the chronic phase; such lesion-based evidence is further supported by functional imaging data derived from healthy participants [95]. In line with these findings, a recent meta-analysis concluded that IPL lesion sites were reversely associated with the performance of patients, with the time post-stroke ranging from >1 month to >12 months [80]. If the IPL is indeed an integral part of a network supporting the cognitive components essential for successful naming, then the shift in lesion–deficit patterns observed in our sample can be explained in the context of neuroplastic changes as discussed above. The question that emerges though is why only the IPL yielded such results. A possible interpretation could be based on the anatomical and functional properties of the inferior parietal cortex. In general, the differential effect of the lesion loci on longitudinal changes related to language recovery could in part be explained on the basis of the cytoarchitectonic characteristics of each brain area [96]. As for the IPL, there are recent studies which suggest that this region is divided into distinct sub-regions with different cytoarchitectonic features. For instance, the study of [97] suggest a significant gender difference in the volume of a sub-region (PFcm) in the supramarginal gyrus. It could be hypothesized then that these brain cytoarchitectonic variations could influence the recovery pattern of different functions, such as naming or auditory comprehension. Moreover, it has been shown that naming ability in healthy older adults is correlated with structural anatomical indices bilaterally, including parietal cortices [98]; this fact, in combination with the proposed laterality shift in the temporo-parietal regions (for a discussion, see [99]) could support the notion that a lesion in the IPL, even though the region may be involved in naming, is less detrimental due to the anatomy-specific highly neuroplastic capacity. In any case, this speculation remains to be confirmed or rejected by future studies with more sophisticated neuroimaging techniques, and probably larger samples.

Overall, our results provide indications about the differential effect of lesion loci on discrete language abilities. The general trend was that specific lesion sites seem to be crucial in the acute but not in the chronic phase. As discussed above, this could be attributed to neuroplastic changes in the context of Goldstein’s theoretical framework about “catastrophic reactions” [100]. Moreover, these findings could provide an explanatory framework for several reports failing to confirm the predicted lesion-to-syndrome correspondence, and thus rejecting the classical thesis of the neo-associationist model; see, for example, [39,40,101]. Another useful insight provided by the present results is that particular lesion loci may be crucial for the recovery of specific language components. For example, the results presented in Figure 6, Figure 7, Figure 8 and Figure 9 indicate that the integrity of the MTG seems to be crucial for the recovery of naming ability, but not for the recovery of comprehension, while the BNT performance seems to improve over time independently of the structural status of the IPL. Nevertheless, these are just speculations which should be further examined in future studies, hopefully overcoming the limitations described below.

One limitation of our study was that related to lesion analysis. While we recruited an adequate number of patients with available CT or MRI scans, the lesion identification method that we used did not allow for a detailed analysis, quantification or specification of the lesion loci and boundaries. Future studies should incorporate more advanced neuroimaging techniques in order to confirm the differential effect of the lesion location on specific language domains at the early or later stages of aphasia. Moreover, the BDAE-SF subscales that we used do not evaluate every aspect of language, such as reading or written word comprehension. The use of this simple language tool is partially justified, mainly because assessing acute patients in clinical practice is rather challenging and demanding. Finally, it is important to note that there was substantial variability concerning the re-evaluation of patients with aphasia. Thus, future studies should test patients using a complex neuropsychological battery and with more detailed imaging techniques, while controlling for confounding factors by implementing a more focused timeframe for the reassessment of stroke individuals.

## 5. Conclusions

In this study, we have showed that specific demographic factors, including age, sex and educational level, do not reliably predict language recovery in patients with aphasia. Notably, we have demonstrated that, depending on the phase, i.e., acute/subacute or chronic, different associations between lesion loci and language functions can be observed. To be more precise, our data indicate that lesions in the middle temporal gyrus influence naming and comprehension only in the chronic phase, while lesions in other traditionally core language areas affect different language functions (speech fluency, naming, comprehension) only in the acute phase. These findings indicate the differential effect of specific lesion sites on language components as a function of time post-onset. Overall, our results highlight the complex and dynamic nature of aphasia recovery and further underlie the need for individualized interventions that consider both lesion location and the evolving neuroplasticity mechanisms in the brain. To that end, future translational studies could investigate the possible neurobiological recovery mechanisms of the different cytoarchitectonic brain areas and how they affect the cognitive procedures of animals and humans, respectively. Also, future research is required to deepen our understanding of the various demographic and clinical factors that could lead to efficient language reorganization in patients with aphasia.

## Figures and Tables

**Table 1:** Demographic characteristics of the patient group.

	Mean	(SD)	Min–Max
**Age in acute phase** (years)	56.45	14.6	23–84
**Education** (years in formal schooling)	11.31	3.6	6–17
**TPO** (in acute phase in days)	17.83	18.6	1–84
**TPO** (in chronic phase in days)	308.8	219.7	42–1154
**Sex**	28 Males 14 Females		

**Table 2:** Performance in language assessment in acute and chronic phase.

	Mean	(SD)	Min–Max
Boston Naming Test (acute)	12.92	14.2	0–44
Boston Naming Test (chronic)	28.03	15.2	0–45
Comprehension BDAE (acute)	21.52	8.7	3–32
Comprehension BDAE (chronic)	27.95	4.8	12.5–32
Speech rate (acute) Speech rate (chronic)	51.41 83.48	41.4 41.7	0–139.53 0–163.64

## Data Availability

Data is unavailable due to privacy and ethical restrictions.
